# Comparison of Machine Learning and Sentiment Analysis in Detection of Suspicious Online Reviewers on Different Type of Data

**DOI:** 10.3390/s22010155

**Published:** 2021-12-27

**Authors:** Kristina Machova, Marian Mach, Matej Vasilko

**Affiliations:** Department of Cybernetics and Artificial Intelligence, Faculty of Electrical Engineering and Informatics, Technical University of Košice, Letná 9, 04200 Kosice, Slovakia; marian.mach@tuke.sk (M.M.); matej.vasilko@student.tuke.sk (M.V.)

**Keywords:** web mining, detection of a troll, machine learning, sentiment analysis, text data processing

## Abstract

The article focuses on solving an important problem of detecting suspicious reviewers in online discussions on social networks. We have concentrated on a special type of suspicious authors, on trolls. We have used methods of machine learning for generation of detection models to discriminate a troll reviewer from a common reviewer, but also methods of sentiment analysis to recognize the sentiment typical for troll’s comments. The sentiment analysis can be provided also using machine learning or lexicon-based approach. We have used lexicon-based sentiment analysis for its better ability to detect a dictionary typical for troll authors. We have achieved Accuracy = 0.95 and F1 = 0.80 using sentiment analysis. The best results using machine learning methods were achieved by support vector machine, Accuracy = 0.986 and F1 = 0.988, using a dataset with the set of all selected attributes. We can conclude that detection model based on machine learning is more successful than lexicon-based sentiment analysis, but the difference in accuracy is not so large as in F1 measure.

## 1. Introduction

The detection of suspicious online reviewers is important in context of revealing antisocial behavior on social networks. The antisocial behavior carried out by trolls and other suspicious reviewers can harm web users and even undermine democracy in many countries. Trolls are involved in spreading hoaxes in serious events such as elections or referendums, so the seriousness of the issue is enormous. The purpose of the work is to offer an effective model for detection of trolls in online space and to join our effort with the effort of many web platforms, which are trying hard today to keep trolls out of business. The significance of the paper is also in comparison of various methods of machine learning and sentiment analysis for the selection of the best method for the detection model building. Thus, the specific scope of this article is to propose different approaches to the generation of a model for troll recognition in various social networks and to compare these approaches. This comparison should give an answer to the question whether it is better to build this detection model using machine learning methods or sentiment analysis methods, and also which specific methods should be used for different types of data—text data and data from the structure of online discussions.

More types of trolls and suspicious authors relate to fake accounts. Fake accounts have become very popular these days, mainly because they permit the manipulation of online web discussions without being responsible. In the work [1] is declared that assessing whether a forum user is harmful or not cannot be made based on one or two posts alone, it is necessary to look at the whole profile. Trolls often hide fake reviews, spam or links to sites containing computer viruses in posts in online discussions. The spam and toxic content can be automatically generated by various systems, for example troll-bots [2]. Web portals are trying to develop automated systems for detecting and blocking suspicious reviewers. Therefore, trolls often mask toxic messages and make them more difficult to detect. Toxic post masking is a technique of writing malicious messages in a way to make difficult for automated systems to recognize them, for example, in a post that is offensive in context, they do not use offensive or rude words. Masking can also be carried out by exchanging letters in some words or by intentional grammatical errors. This requires the creation of very rich, expressive, and carefully created lexicons [3].

The main categories of trolls are provocative troll, professional troll, disseminator of disinformation, troll opponent and social-engineering troll [4]. One troll may have many false accounts and in an online discussion he(she) supports own contributions to give them the necessary credibility and thus efficiency in influencing public opinion through misinformation. Trolls often publish fake articles that refer to non-existent, fabricated scientists. Recently, so-called troll farms have emerged, which are much more dangerous than a single troll. It is an organized group of trolls that usually receive some form of compensation for their activities, such as financial reward. This coordinated group of tens of trolls can effectively create the illusion of bigger support for its sponsor. Troll farms are thus used to improve (or worsen) the online “image” of somebody on social networks [4]. In 2019, the “freedomhouse.org” portal provided extensive research called “Freedom on the Net” to uncover trolls and identify a frequency of their appearance in online policy-related discussions. The result has been an extensive report claiming that troll farms are currently used in top politics in probably as many as 30 countries around the world [5].

There are more approaches, how to detect troll poster. It depends on a discussion forum, where he/she is posting and on the type of data, which can be extracted from this forum. If only text data are available, *natural language processing* can be used to analyze the dictionary and the style typical for troll poster. Another approach is *machine learning*. This approach is more universal as it can process both text data with attributes in the form of words but also other type of data with such attributes as number of likes/dislikes, number of posts, number of responses and so on. One of the problems with this approach is that accounts on different social networks provide different information about user accounts and this leads to different attributes and prevents learning sufficiently general models. Models based on machine learning methods are therefore often designed for specific platforms [4].

The paper [6] presents results of more machine learning methods using data from the platform Twitter. The work focuses on detection of real profiles of suspicious authors and on fake profiles created to cover up antisocial behavior. The following data about each profile has been extracted from Twitter: *texts of posts* (texts can be helpful for indication of a writing style from the post texts, whether it uses offensive words, bad or vice versa the correct grammar, colloquial expressions, or the presence of hoaxes or hot themes), *date and time of publication* (trolls tend to usually write posts at short intervals of time to effectively attack the discussion), *language and geographical position of the profile* (can be helpful in tracing of a fake profile), *number of followers*, and *twitter client* (while the ordinary user contributes with several devices, the troll uses usually the same device). For a model training were used the following methods of machine learning: random forest (RF), algorithm J48, K-NN, sequential minimal optimizer (SMO) and naïve Bayes. The best results were achieved by the RF (Accuracy = 0.665) and SMO (Accuracy = 0.685).

The paper [7] presents results of model training on data from the platform Reddit. Since the Reddit contains many malicious and suspicious posts, a team from the scientific portal Towards Data Science tried to develop a system using machine learning methods that will detect suspicious accounts on this web platform. The system is primarily focused on political trolls. The first step in creating the system was to create an information bar that allows moderators to check and potentially remove comments that appear in real time. The architecture of this bar is based on data streams management in the Apache Kafka software platform, which makes it easy to work with real-time data streams and facilitates data processing. In addition to the *text of the post*, it is possible to obtain such data as *account name, date and time of submission, number of comments and reactions* to that post or *reddit-karma* of the author. A machine learning method for generating decision trees (DT) was used to train the model. The system used data on 93,668 users of the Reddit discussion forum. This data was divided into a training and testing set in a ratio of 70% to 30%. The model learned to recognize classes: troll, chatbot and regular user. The model achieved for a troll recognition F1 = 0.702, for a chatbot recognition F1 = 0.899, for regular user F1 = 0.947 and an overall Accuracy = 0.917.

**Figure 1:**
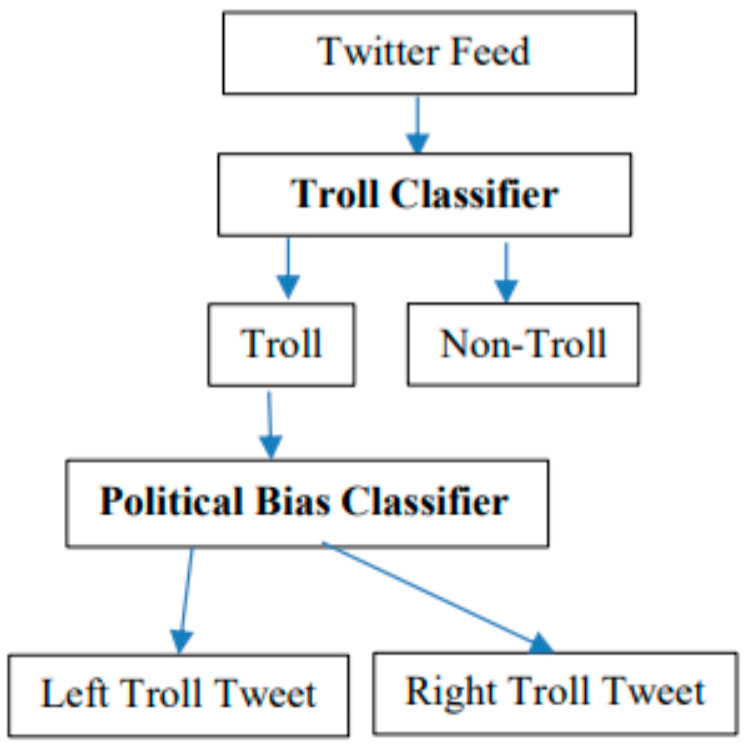
Process of classification consists of two steps. At first step, the troll tweets are recognized using “Troll Classifier”. In the second step, the “Political Bias Classifier” is used to recognize political orientation of previously recognized troll tweets, which can be left (Left Troll Tweet class) or right (Right Troll Tweet class) [8].

Another work [8] used data from the platform Twitter to train a model titled Political Bias Detector. This detector classifies trolls according to their political orientation on right-wing and left-wing trolls. It consists of two classifiers the Troll Classifier and the Political Bias Classifier, as you can see in Figure 1.

Achieved results were following:

- SVM (Support Vector Machine)—Accuracy = 0.84 for troll/non-troll classification, Accuracy = 0.85 for classification of right-wing or left-wing troll;
- Convolutional neural network—Accuracy = 0.74 for troll/non-troll classification, Accuracy = 0.84 for classification of right-wing or left-wing troll;
- BERT (Bidirectional Encoder Representations for Transformers) model—Accuracy = 0.99 for troll/non-troll classification, Accuracy = 0.89 for classification of right-wing troll or left-wing troll.

The neural networks were used also in work [9]. Weller and Woo from Stanford University developed a system for troll detection using deep learning on data from portal Reddit. They used architecture with 4 layers: BERT—pre-trained embedding layer, neural hidden layer, dropout layer and classification layer. The hidden layer contained convolutional and recurrent LSTM layers. The purpose of the Dropout layer was to ignore randomly selected neurons during the training phase. This limits the interdependence between neurons by ignoring some of their connections. The classification layer was an output layer that used Softmax activation function. Model based on recurrent LSTM achieved Accuracy = 0.727 and model, which used *convolutional layers*, achieved Accuracy = 0.756.

Machine learning methods can be used also in solving the sentiment analysis problem and consequently the sentiment analysis can be helpful for troll detection. The work [10] proposes a multimodal framework for Persian sentiment analysis. Multimodality of the approach is represented by processing text data as well as acoustic and visual data. The work showed that multimodal features delivered better performance (Accuracy = 0.914) than unimodal visual features (Accuracy = 0.892). When only text data from hotel reviews were used [11], CNN achieved Accuracy = 0.823 and LSTM model had Accuracy = 0.850. This work has integrated grammar-based rules and neural networks.

The article [12] introduces semi supervised learning of detection models for spam reviews. Machine learning methods were combined with time series. The suspected time intervals were captured from a time series model of all the reviews. Then semi supervised learning in each captured interval was provided to obtain a spam author detector. They achieved the best result F1 = 0.82 using their approach. In [13], there is introduced an approach for learning a prediction model for web user rating using deep learning from textual reviews collected from Yelp. The unreliable reviews were detected via their bad ratings using several deep learning kernels. It was not solved as classification task.

On the other hand, work [14] is not focused on detection of troll or spam reviewers, but on detection of an uncreditable and fraudulent activity of web users, so called phishing. It uses surprisingly K-Nearest Neighbour (KNN) machine learning method for detection phishing attacks provided via url classification. K = 10 is the value that gives the best Accuracy = 0878 on phishing attack detection trained on data from Kaggle. Overall, the average accuracy of the proposed model is 0.858.

The work [15] tried to solve the problem of spam reviewer detection. Authors at first learned an unsupervised deep aspect level sentiment model using Boltzman machines to discriminate genuine reviewers, which usually focus on important aspects of entities, from spam reviewers providing more text but on less important aspects. At second, LSTM network was trained to track the evolution of opinions in a temporal context because spammers usually target the specific short time period to cause maximum bias to the public opinion.

Paper [16] focuses on detection of reputation fraud campaigns in product review data streams. Authors monitored online reviews using dynamic programming to generate the most abnormal review subsequences and then they exploited conditional random fields to label a review as suspicious or genuine. They provided extensive experiments to evaluate the Fraud Guard model.

For these types of problems, using neural networks is today a preferable approach to represent detection models for suspicious authors recognition. For learning of neural network parameters various optimization methods are used. It is possible to use traditional methods (e.g., Stochastic Gradient Descent) or some newly developed optimization method (e.g., method developed in paper [17]).

## 2. Materials and Methods

### 2.1. Data Description

Data from a Reddit discussion forum was obtained for this work. The Reddit is gaining increasing popularity, but more and more suspicious users are emerging there. Every year, Reddit provides data on suspicious accounts and suspicious comments for scientific purposes. For the purpose of this work, 6695 records of trolls were selected, which were stored in a separate dataset. We also obtained data from this portal: 10,000 records of ordinary users who were not trolls. The data were extracted exclusively from online discussions on political topics.

The troll data contained several attributes that did not appear in the data on ordinary users. Therefore, it was necessary to reduce the set of attributes so that the attributes matched and thus the data set of trolls could be combined with the data set of ordinary users. In this way a final labelled set was formed, where label was represented by attribute *Is_troll* (value1 indicates that the user is troll, value of 0 indicates that he is an ordinary user). All extracted texts in English were put into attribute *Body*.

These texts were processed using the Python-implemented *Afinn* library, which provides the ability to analyze sentiment of a text. Based on lexicon-based sentiment analysis, numeric values representing the degree of positivity or negativity of the text were assigned to all comments. The current version of the lexicon of this library-*Afinn en − 165* contains more than 3300 words in English. Each of these words has a polarity value associated with it. The resulting comment polarity value was determined by summarizing the polarities of the words in the text if they matched the words in the lexicon. This library also allows the intensification and processing of negation in the form of “switch negation” as well as with combined negation. It can also process frequently used smile emojis. This library was chosen because it submitted reliable results after testing on selected comments.

A positive polarity value of a sentence represents that the given text is positive in terms of polarity, while a negative value expresses that the text has negative polarity. A value of zero indicates that the text is neutral. If a comment has an overly negative rating (for example −15 or −20), the comment can be considered toxic, since to achieve such negative ratings, a comment must contain vulgarisms, offensive words or insults. Since the work deals with users in political conversations, when revealing trolls, it is necessary to focus on comments that have either highly negative or even highly positive polarity. Neutral comments don’t play such a prominent role in revealing a troll. These values of polarity of comments generated by the library *Afinn en − 165* created the values of another attribute titled *Score*. After a closer examination of the rated comments, it can be seen that comments from troll users actually tended to have more negative or more positive reviews than those of ordinary users. The comments of ordinary users took the ratings closer to zero, i.e., neutrality. From all considered attributes, we have chosen the following final list of attributes:

- *Is_troll*—labelling
- *Score*—opinion polarity (described above)
- *Ups*—is the number of likes received by the user on his/her posts on the Reddit portal.
- *Down*—is the number of dislikes received by the user on his/her posts and comments.
- *Link_karma*—this attribute is like the *Comment_karma*. However, *Link_karma* attribute does not reflect the karma of the comments, but the karma of published posts of the user.
- *Comment_karma*—represents the karma of the user’s comments on the Reddit forum. If the user is too toxic, posts troll comments, or adds hoaxes, it can be assumed that the karma of his comments will not be too high.
- *Has_verified_email*—indicates whether the user has a verified email address. Each user must enter an email address at the time of registration, to which the confirmation email arrives. If someone does not confirm this address, it could indicate that this author created the fake profile only to write for example troll comments and no longer handled this account.
- *Is_gold*—is a binary attribute with values 1 and 0. A value 1 means that the user account has purchased a premium membership. It can be assumed that a user who has a premium membership is less likely to be a troll, as premium membership on this portal is subject to a fee.
- *Controversiality*—on reddit, moderators regularly refer to hoaxes or controversial posts. The *Controversiality* attribute indicates that the user has already had a post assessed as controversial in the past. If a troll has been using his troll account for a long time, it is possible that one of the moderators has already rated some of his post or comment as controversial.
- *Is_mod*—indicates whether the user is a moderator of the Reddit discussion forum. It can be assumed that the moderator will not be a troll.

Within pre-processing, records with many missing values have been deleted. We have supplemented missing value with the average value of the given attribute when the record had only a few missing values. The final dataset titled “Final_Data_New.csv” is available at people.tuke.sk/kristina.machova/useful/. Data in “Final_Data_New.csv” obtain, in addition to the attributes described above, attribute “*Edited*” (how often the reviewer edited his comments—the value was the number of edits or “false” if he never edited his comments) and attribute “Num_comments” (number of comments of the reviewer or average value from numbers of all reviewers equal to 484 for missing values). Attributes Edited and Num_comments were not used for training finally, because they contained too many missing values.

For creation a CNN model and an application for a troll detection based on sentiment analysis only data with attributes *Is_troll* and *Body* were used. The text data consist of two parts “Is_troll_body.csv” and “Is_not_troll_body.csv” and are available at people.tuke.sk/kristina.machova/useful/.

### 2.2. Used Methods

We have used methods of machine learning as well as lexicon-based sentiment analysis. Machine learning approach seems to be a good alternative according to related works and according to our experiences with building models for detection of various forms of antisocial behavior.

The state-of-the-art shows that machine learning methods such as RF, J48, K-NN, SMO in detecting trolls, trollbots and fake accounts are not such a good choice. Better results were achieved with DT, neural networks in particular CNN. That’s why we focused on NB as a baseline method and on DT, SVM, forward NN and CNN. Our goal in the building detection models for troll recognition was also to increase the efficiency of machine learning models compared to the results achieved in related works.

From our experiences, the best model for recognition of fake posts was learned using Naïve Bayes (NB) classifier from extremely short texts [18]. SVM and NB methods have proven successful recognition of trolling posts [19] and deep learning (DL) in toxic comments classification [20].

#### 2.2.1. Machine Learning Approach

Based on the above, we decided to focus on the following methods of machine learning: Naïve Bayes classifier, support vector machines, decision trees, and neural networks.

Naïve Bayes classifier (NB) is a probabilistic classifier based on Bayes’ theorem and independence assumption between features. Naive Bayes is often applied as a baseline method; however, its performance is reported to be outperformed by support vector machines [21].

Support vector machines (SVM) separates the sample space into two or more classes with the widest margin possible. The method is originally a linear classifier; however, it can relatively efficiently perform non-linear classification by using a kernel function. Continuing to complete the solution (creating the widest margin between samples), it was observed that only nearest points to the separating hyperplane determine its margin. They are called support vectors. The objective is to maximize the distance of these support vectors from the hyperplane, which is known as the primal problem of support vector machines [22].

Decision tree (DT) represents a model which uses a tree of decisions to predict a label for a new sample. Assuming a standard top-down approach, method starts with a full dataset in one root node. A generated question divides a node to sub-nodes each representing one possible answer to the question. Focusing on subsets, they are generated according to a class diversity. There are two most used types of diversity functions, Information Entropy and Gini index [23]. Best ranked questions generate minimal disorder. The advantages of decision trees are their intuitive interpretation and non-linear characteristics.

Feed-forward neural networks (FNN) consist of a finite number of layers that contain finite numbers of neurons. There are no feedbacks backwards between layers or between individual neurons. The number of neurons should be sufficient to solve the problem, and the number of layers should be minimal to reduce the time for the problem solving. Convolutional neural networks (CNN) became more popular in 2012, however, they originated in the work Generalization and network design strategies [24]. CNN has topology defined by three types of layers: *convolutional layer*, *pooling layer*, and *fully connected layer*. The *convolutional layer* is represented by a set of kernels.

#### 2.2.2. Sentiment Analysis Approach

Sentiment analysis can be provided using again the machine learning approach and a lexicon-based approach. There are some works, which have used machine learning approach to sentiment analysis for example [25] have developed Ensemble Learning Scheme using DT, SVM, RF and KNN (K-Nearest Neighbours) for sentiment analysis of COVID-19 related comments. In work [26] deep learning models for sentiment analysis were used in recommender systems. There are some related works using sentiment analysis based on machine learning for developing applications for recognition of trolls. For example, work [27] uses sentiment analysis based on SVM and XGBoost (boosting method of machine learning for learning a set of models) for troll recognition. They achieved best accuracy over 0.82 using XGBoost. In addition, work [28] uses machine learning method—particularly NNS (Nearest Neighbours Search) for troll detection by sentiment analysis.

We have used lexicon-based approach to sentiment analysis instead of using machine learning approach for distinguishing trolls from common users of online discussion. For our purpose it is more suitable to select the lexicon-based approach because it can better detect dictionary typical for trolls. Effectivity of lexicon approach, of course depends on the correct choice of words in the lexicon and their most accurate assessing with the degree of polarity. In our work [29] the effective lexicon-based application for sentiment analysis is described. For this application two lexicons were generated-Big and Small. The Big lexicon (domain-depended) was translated from English and enlarged by domain-dependent words, to increase its effectiveness. The Small lexicon (domain-independent) was extracted from six English lexicons when only overlapping words from all lexicons, which were domain-independent, were included. Both lexicons were labelled using optimization method PSO (Particle Swarm Optimization) more precisely BBPSO (Bare-Bones PSO). The application effectivity was increased also by intensifications processing and combined negation processing, when two approaches, “switch” and a “shift” negation processing, interactively cooperate. This application was used for the sentiment analysis, more precisely for analysis of an opinion polarity of posts of suspicious reviewers—trolls.

Our approach to troll-opponent recognition is based on an idea, that this kind of troll has tendency to express opposite opinion as the opinion of whole online discussion and the troll’s opinion is usually extremal. Therefore, the posts of the investigated suspicious reviewer and the posts of all other participants in the online discussion are extracted separately from the discussion. Subsequently, using our lexicon-based opinion analysis application, the average degree of polarity of the suspicious reviewer’s opinions against the average degree of polarity of the entire remaining online discussion is determined.

The average polarity is important for comparison. Some author can produce a great number of posts and another author only a small number of posts. We can use sentiment analysis to find the values of polarity of all posts. If we use simple summary of polarities of all posts of some author, author with many posts will have greater summarized value of polarity, but it will be not very precise expression of his opinions. We must therefore divide the summary of all the polarities of individual posts by the number of posts. Thus, the summary value of the polarity will represent the average value from interval of integers <−3, +3>, and will be more suitable for comparison with the polarity of the whole discussion, which will also be the average value from <−3, +3>.

Then it is calculated a difference between the average degree of polarity of suspicious reviewer and of the whole remaining discussion. If this difference exceeds a predetermined threshold, then the given reviewer is classified to “Troll” class and if it is below the threshold, then the reviewer is classified to “Non-troll” class. This Threshold was set to 2 experimentally, considering, that polarity values are represented as integers from the interval <−3, +3>.

**Figure 2:**
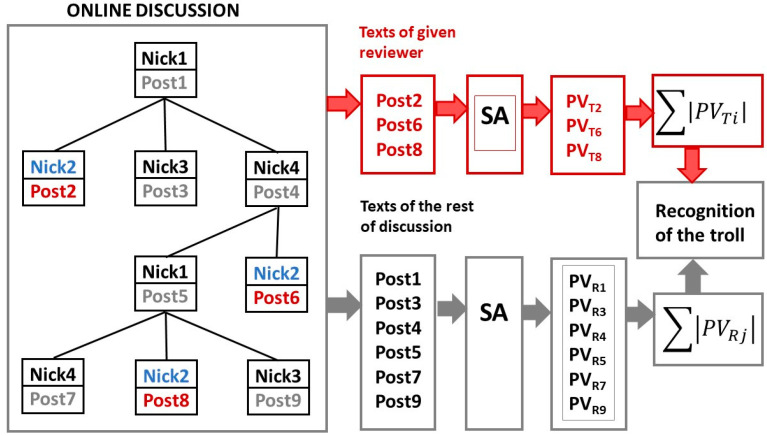
Illustration of the new approach to detection of the *TROLL_opponent* using an example with four reviewers in online discussion. The opinion polarity values of posts 2, 6 and 8 of reviewer Nick2 are compared to the opinion polarity of the other posts 1, 3, 4, 5, 7 and 9 in the online discussion. Particularly, the average absolute value of polarity of all posts of the examined reviewer is compared to the average absolute value of polarity of all rest posts of the online discussion. If the difference between them is greater than given threshold (see Equation (1)), then the examined reviewer is classified to the class *TROLL_opponent*.

Experiments have shown that calculating the average polarity described above may not be the best. It can also happen that the *TROLL_opponent* opposes really everything, regardless of whether the original comment is positive or negative. The *TROLL_opponent* reacts usually very negatively to the positive and, on the contrary, although in the same discussion he reacts very positively to the negative comments. Thus, the average polarity of his comments can be neutral (close to zero). It was therefore necessary to modify our approach and work with the absolute average polarity value. The differential is then calculated as the average of the polarities of all the comments of one reviewer, but with the negative values reversed to positive. It means, only absolute values of polarities are considered. This method of calculating the average polarity must be then reflected also in the computing of the polarity of the whole discussion. This method can be modeled by Equation (1). The illustration of this method is in Figure 2.

(1) $$IF\;\sum^{\;}\left|PV_{Ti}\right|-\sum^{\;}\left|PV_{Rj}\right|>Threshold\;Then\;TROLL\_opponent$$

Parameter *PV_Ti_* is the average polarity value of texts of examined reviewer-probable Troll (index *Ti*) and *PV_Rj_* is average polarity value of the other texts of the given online discussion—the rest texts (index *Rj*). The opinion polarity values were obtained using our lexicon-based application of SA (sentiment analysis). In Figure 2, the examined reviewer is Nick2. His texts (Post2, Post6 and Post8) are compared with the other texts of discussion from the point of opinion polarity

## 3. Results

### 3.1. Classification Using Explainable Methods of Machine Learning

We have focused at first on classic methods as naïve Bayes, decision trees and support vector machines. These methods generate models, which can be intuitively well explained. Naïve Bayes offers conditional probabilities, which represent measure of belonging attributes to classes for recognition. Decision trees are well known as they are understandable and explainable. Support vector machines are often used because of they form a mathematical model of hyperplane dividing two classes. The model contains information about the importance of particular attributes in it. The baseline method for experiments was Naïve Bayes Classifier. We have run three experiments on dataset “Final_Data_News.csv” available at people.tuke.sk/kristina.machova/useful/. The dataset was divided using ratio 60% (training set) to 40% (test set) and used for training machine learning models. More experiments were provided with a few different subsets of attributes. Those sets of attributes are presented in Table 1.

In the first Set 1 of attributes, we took into account primary attributes that did not contain any missing values in the records in the dataset. In Set 2 we included all attributes that were not used in the first experiment (*Controversiality*, *Link_karma* and *Is_mod*). On the other hand, we missed the attributes *Has_verified_email* and *Downs*. The *Has_verified_email* is assigned value 1 much more often than 0. We omitted the *Downs* attribute because not always a user with a large number of dislikes must be a troll and this attribute might be misleading. For the evaluation of models’ efficiency, we have used well known measures (Accuracy, F1 score, Precision, Recall and Specificity) but also Matthews Correlation Coefficient (*MCC*). The values of *MCC* were calculated by Equation (2).

(2) $$MCC=\frac{TP*TN-FP*FN}{\sqrt{\left(TP+FP\right)\left(TP+FN\right)\left(TN+FP\right)\left(TN+FN\right)}}\;,$$

where *TP* represents a number of true positive classifications, *TN*—true negative, *FP*—false positive and *FN*—false negative. Achieved results of Naïve Bayes model for all three sets of attributes (Set 1, Set 2 and Set 3) are present in Table 2. 

The accuracy in all experiments was higher than 60%, what is not a very big success. Only recall was high—over 90% in all experiments. It means, that the number of false negative classifications are very low. However, on the contrary, the of false positive classifications are very high. That represents a model, which is good in recognition of non-troll user, but not very good in recognition of troll users. However, this is the first basic model. The highest achieved Accuracy = 0655 was achieved using the smallest set of attributes, where 3 attributes were being excluded.

We have learned also the detection model using SVM on data with primary attributes (data not containing any missing values—Set 1). At first, we experimented with various SVMs—a linear SMV, the SVM with Gaussian Kernel Radial Basis Function (GKRBF) and the SVM with Sigmoid Kernel Function (SKF). The results are in Table 3.

Results in Table 3 showed, that from these three various SVM methods the best method to learn a most effective detection model for troll recognition is SVM + GKRBF. So, we focused particularly on this method when trying to learn recognition models on all three sets of attributes introduced in Table 1. Achieved results of SVM + GKRBF for all three sets of attributes are presented in Table 4. 

In the case of the SVM method the model trained on the whole set of attributes (Set 3) achieved the best results. However, the differences of effectivity of these three models learned on three sets of attributes were not very large and significant. We can say that this model is excellent in all measures.

Another model for troll recognition was learned using decision trees (DT). DT models are not usually very good on text data, but our data in Set 1–Set 3 are not text data and this model is of a very intuitive and explainable type. Achieved results of Decision Trees model for the three various sets of attributes are present in Table 5. Results achieved by DT model are surprisingly good, even if they did not overcome the effectiveness of the SVM method. The best results were achieved in the third experiment on data with full set of attributes. The differences between Precision and Recall are not so big as in NB model. It means approximately similar numbers of false positive and false negatives classifications. 

### 3.2. Classification Using Neural Networks

In several works, neural networks (NN) provide reliable results in the classification of trolls. For NN, in addition to the training and testing set, a validation set has also been created. We used Tensorflow and Keras libraries, which are implemented in Python. Since the attributes are numeric or binary, we used a forward neural network with hidden layers.

We used sequential linking of individual layers, which are represented by the object Sequentional(). The design of NN contains an input layer, five hidden layers, and an output layer. We wanted to compare the results of the NN models with the best models of other machine learning methods. It was the reason that models were taught on Set 3 of attributes, so all attributes were used in experiments with NN. In all three experiments with NN, we did not change the set of attributes but the structure of the NN (see Tables 6, 8 and 10). Table 6 describes an architecture of NN1 model in the first experiment. In this experiment dropout regularization, Adam optimization and 50 epochs were used in the process of NN1 training.

**Figure 3:**
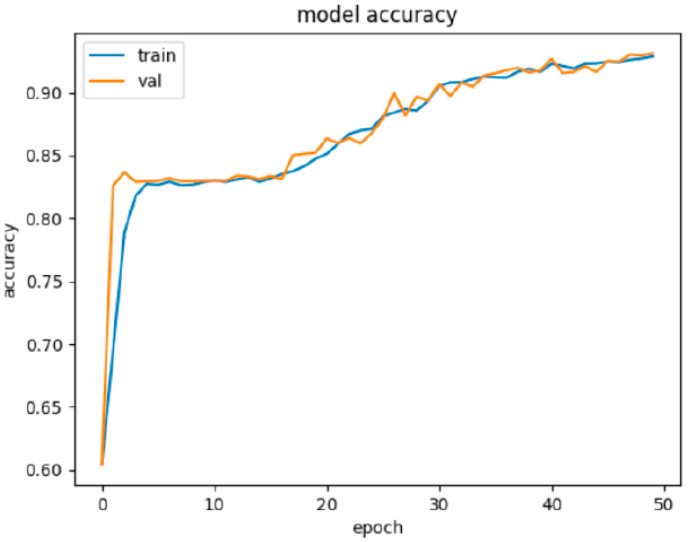
Graphic visualization of the progress of NN1 learning on the training and validation set. We can see that learning was very effective in the first few epochs and accuracy above 80% was achieved very quickly. This was followed by a phase of stagnation of the learning process up to the breakthrough somewhere around the 17th epoch, when accuracy began to grow again and achieved over 90% of accuracy after 50 epochs.

The results of this experiment are presented in Table 7. Figure 3 illustrates the learning progress on the training and validation set. Results in Table 7 were achieved on the testing set.

When using all attributes, this network achieves very good results. This experiment showed that the neural network can achieve better results than NB and DT. On the other hand, SVM results are better than those achieved with this NN1 model, so we tried to train better NN model. In the next experiment, all attributes were used again, but with the neural network structure changed as is described in Table 8. 

**Figure 4:**
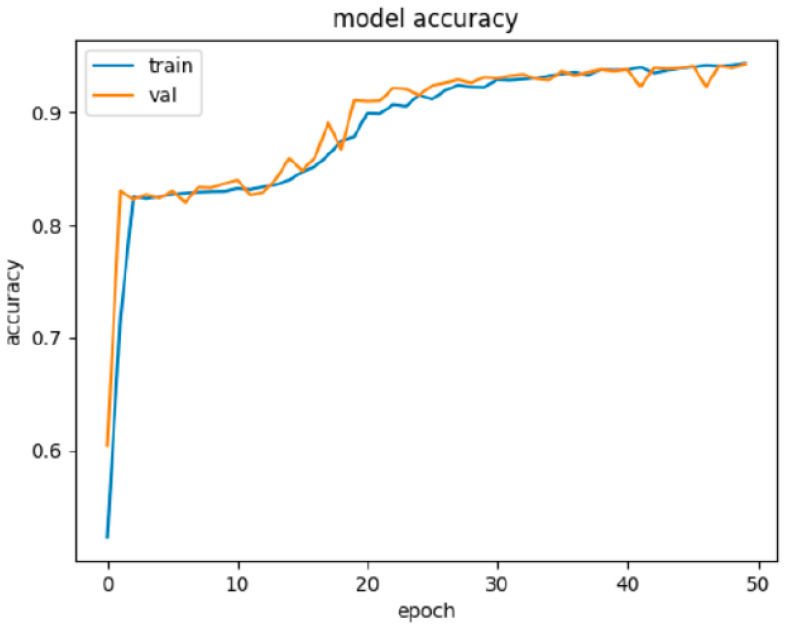
Graphic visualization of the progress of NN2 learning on the training and validation set. In the case of NN2, the learning was again very effective in a few first epochs and accuracy nearly 84% was achieved very quickly. This was followed by a phase of stagnation which was shorter than stagnation in learning NN1. After stagnation of the learning, accuracy began to grow again from the breakthrough somewhere around completing 12 epochs and achieved over 94% of accuracy after 50 epochs.

The results achieved on the testing set in the second experiment with structure of NN2 (described in Table 8) are presented in Table 9. The model NN2 was trained at 50 epochs. We have used Dropout regularization and optimization method Adam to regulate learning. Figure 4 illustrates the learning progress on the training and validation set.

### 3.3. Recognition of Trolls from Texts Using Sentiment Analysis

We have managed to improve the results of the NN1 by using the new structure in the NN2 model, but the NN2 model still does not overcome the results of the SVM + GKRBF model.

In our last experiment with NN, we have used only attribute *Body*, which contained texts of posts. The text data consist of two parts “Is_troll_body.csv” and “Is_not_troll_body.csv” and are available at people.tuke.sk/kristina.machova/useful/. The aim of this experiment was to train a model able to recognize a troll only using texts of his posts. For texts processing is more suitable convolutional neuron network (CNN). 

As the first step in the pre-processing process, it was tokenization of the texts in the dataset using the *tokenizer* method that is implemented in the *keras* library. The comments were pre-processed into a matrix form in which each word is represented by a unique number. This was followed by the training of a neural network with convolution layers. The architecture of CNN is described in Table 10. The results are in Table 11.

The results of CNN model are the best from results of all neural networks models, but still not better than SVM model. The results of the different efficiency measures are balanced, which means that the numbers of false positive and false negative classifications are approximately the same and very small.

### 3.4. Recognition of Trolls from Texts Using Sentiment Analysis

Our new approach to the recognition of troll-opponent based on lexicon sentiment analysis was implemented in the programming language *Java* in the development environment IntelliJ. Sentiment analysis was used to compare the opinion polarity of suspicious author to the opinion polarity of the whole online discussion. Big difference between these two opinion polarities can lead to troll-opponent recognition. The achieved results are referred in Table 12.

The results of model for troll recognition based on sentiment analysis are good, but not better, than results of CNN model. This model has also higher Recall then Precision. It means that it is better in searching for all possible trolls, but more from them are not trolls really.

## 4. Discussion

**Figure 5:**
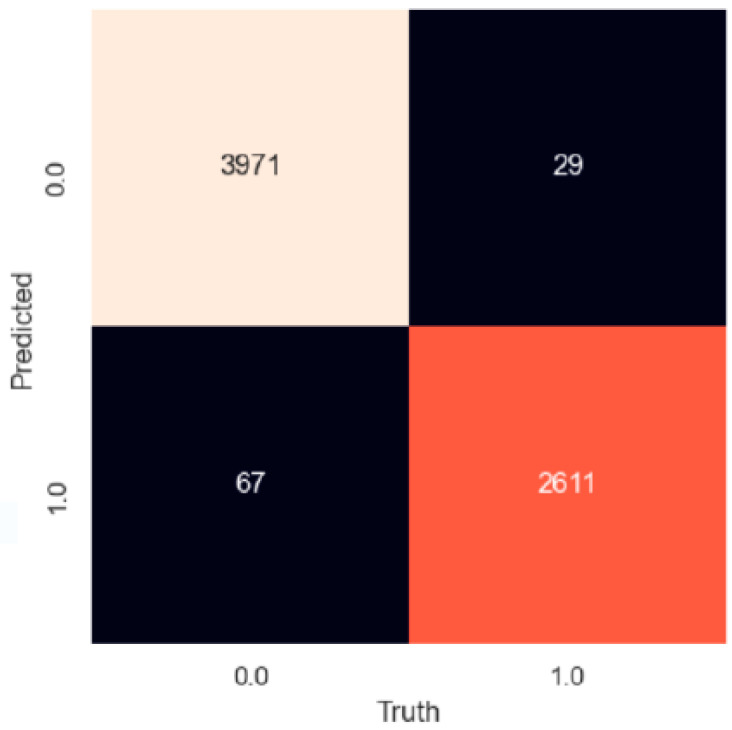
Confusion matrix for the best model for troll detection trained by SVM + GKRBF.

We have trained detection models for recognition of the troll contributors to online discussions using different machine learning methods but also using sentiment analysis based on the usage of a lexicon. The models were trained on text data (attribute *Body*) and on non-text data (attributes *Is_troll*, *Has_verified_email*, *Is_gold*, *Controversiality*, *Comment_karma*, *Link_karma*, *Score*, *Ups*, *Downs*, *Is_mod*) extracted from online space. For the purpose of comparison of all used methods, the best results of all trained models are presented in Table 13. The best results achieved on a different type of attributes what means on different type of data are presented in bold. The confusion matrix for the best model at all is in Figure 5.

According to Table 13 the best result on non-text data was achieved by SVM with Gaussian Kernel Radial Basis Function. This method significantly outperformed baseline method NB and easily outperformed DT. DT model was pleasant surprise for us as we did not expect such good results with this method. SVM + GKRBF model was slightly better than models trained with Feed-forward NN.

On text data, two different approaches were used—machine learning approach (CNN) and lexicon-based sentiment analysis approach (LSA). The CNN model outperformed the approach based on comparison of summarized opinion of reviewer and all online discussions using lexicon-based sentiment analysis. For comparison our approaches with relation works, Table 14 was created. The table contains only best results of each approach. We can see that our approaches outperform all related works except BERT. We can see that SMO models in [6] achieved poor result similar to our NB model. On the other hand, DTs in [7] were slightly better than our DT model. However, our SVM model was by more than 10% better than in [8]. Neural networks models in [8,9] have been outperformed by our CNN by more than 20% of accuracy. It has been shown that it is preferable to use a lexical approach (our LSA) than a machine learning-based approach in [11] when using sentiment analysis to detect trolls.

Our proposed approach is limited by the use of data of different types. Some methods of generating a detection model are more successful on text data and others on data from usage and structure of discussion. Text can be very well processed by deep learning, but deep learning methods often require overly large amount of data for training, which is not available. Perhaps it would be appropriate to explore in future the possibilities of learning the model from mixed both text and non-text data. Another way may be using meta-learning or learning by a set of different methods. Another limitation of our approach is connected with pre-processing. Pre-processing of text data should be simple to avoid the loss of specific information typical of trolls (intentional errors, capital letters, slang words, abusive dictionary, etc.). On the other hand, data from structure and usage need more sophisticated pre-processing.

The challenge for related applications of the current work could be extraction of data from live an online discussion to search trolls in it. For all reviewers, all texts should be extracted and also the exact values of all attributes from the structure and usage. All the information will be needed to discriminate trolls from common users in the search online discussion. This automatic extraction should be provided in real time every time, when the application is started.

Future research directions may be in extended experiments involving Bidirectional Encoder Representations for Transformers. Another challenge for future is to recognize the offensive speech from combination of text data and non-text data, what is not a trivial goal, because some methods are excellent in text data processing (for example neural networks with memory) but in case of non-text data different types of machine learning methods are more successful (for example SVM).

## Figures and Tables

**Table 1:** Sets of attributes used for learning models using machine learning methods.

Attributes	Set 1	Set 2	Set 3
*Is_troll*	**+**	**+**	**+**
*Has_verified_email*	**+**	**-**	**+**
*Is_gold*	**+**	**+**	**+**
*Controversiality*	**-**	**+**	**+**
*Comment_karma*	**+**	**+**	**+**
*Link_karma*	**-**	**+**	**+**
*Score*	**+**	**+**	**+**
*Ups*	**+**	**+**	**+**
*Downs*	**+**	**-**	**+**
*Is_mod*	**-**	**+**	**+**

**Table 2:** Effectivity of Naïve Bayes model for recognition of troll users in online discussions.

Measures	Set 1	Set 2	Set 3
Accuracy	0.655	0.615	0.627
F1 score	0.611	0.541	0.545
Precision	0.453	0.379	0.391
Recall	0.938	0.948	0.938
Specificity	0.539	0.511	0.516
Matthews Correlation Coefficient	0.409	0.399	0.402

**Table 3:** Effectivity of models learned by linear SVM, SVM with GKRBF and SVM with SKF for recognition of troll users in online discussions.

Measures	Linear SVM	SVM + GKRBF	SVM + SKF
Accuracy	0.738	0.974	0.727
F1 score	0.762	0.978	0.769
Precision	0.697	0.973	0.780
Recall	0.839	0.983	0.758
Specificity	0.839	0.960	0.663
Matthews Correlation Coefficient	0.485	0.945	0.429

**Table 4:** Effectivity of models learned by SVM with GKRBF on three various sets—Set 1, Set 2 and Set 3 of attributes from Table 2.

Measures	Set 1	Set 2	Set 3
Accuracy	0.974	0.984	0.986
F1 score	0.978	0.987	0.988
Precision	0.973	0.983	0.992
Recall	0.983	0.981	0.983
Specificity	0.960	0.989	0.989
Matthews Correlation Coefficient	0.945	0.967	0.970

**Table 5:** Effectivity of Decision Tree models for recognition of troll users in online discussions.

Measures	Set 1	Set 2	Set 3
Accuracy	0.789	0.849	0.901
F1 score	0.788	0.882	0.922
Precision	0.653	0.929	0.875
Recall	0.995	0.831	0.954
Specificity	0.657	0.887	0.835
Matthews Correlation Coefficient	0.650	0.685	0.805

**Table 6:** Architecture of NN1 model in the first experiment (Adam optimization, 50 epochs).

Layer	Parameters	Activation Function
Input	9 neurons (9 attributes)	—
Dense 1	55 neurons	Sigmoid
Dense 2	200 neurons	Sigmoid
Dense 3	100 neurons	ReLU
Dense 4	20 neurons	Sigmoid
Dropout	rate 0.2	—
Dense 5	11 neurons	ReLU
Output	2 (binary classification)	Sigmoid

ReLU (Rectified Linear Unit).

**Table 7:** Effectivity of the forward NN1 model for recognition of trolls learned on Set 3 (all attributes). The model was trained at 50 epochs.

Measures	Set 3
Accuracy	0.934
F1 score	0.945
Precision	0.954
Recall	0.937
Specificity	0.929
Matthews Correlation Coefficient	0.862

**Table 8:** Architecture of NN2 model in the second experiment.

Layer	Parameters	Activation Function
Input	9 (9 attributes)	—
Dense 1	80 neurons	Sigmoid
Dense 2	300 neurons	Sigmoid
Dense 3	200 neurons	ReLU
Dense 4	100 neurons	Sigmoid
Dense 5	50 neurons	Tanh
Dropout	c 0.2	—
Dense 6	20 neurons	Sigmoid
Output	2 (binary classification)	Sigmoid

**Table 9:** Effectivity of the forward NN2 model for recognition of trolls learned on Set 3 (all attributes). The model was trained at 50 epochs.

Measures	Set 3
Accuracy	0.947
F1 score	0.956
Precision	0.955
Recall	0.956
Specificity	0.933
Matthews Correlation Coefficient	0.889

**Table 10:** Architecture of CNN model trained on text data.

Layer	Parameters	Activation Function
Embedding	20,000 input dimension, 200 output dimension, 100 input length	—
Convolutional 1	29 filters (size 3)	ReLU
Convolutional 2	9 filters (size 3)	ReLU
Maxpool	—	—
Dense	50 neurons	Sigmoid
Output	2 (binary classification)	Sigmoid

**Table 11:** Effectivity of the CNN model for recognition of trolls.

Measures	Set 3
Accuracy	0.959
F1 score	0.965
Precision	0.963
Recall	0.968
Specificity	0.945
Matthews Correlation Coefficient	0.914

**Table 12:** Effectivity of the model for recognition of trolls based on lexicon sentiment analysis.

Measures	Set 3
Accuracy	0.950
F1 score	0.811
Precision	0.769
Recall	0.833
Specificity	0.966
Matthews Correlation Coefficient	0.772

**Table 13:** Effectivity of models learned by all used methods for recognition of troll users in online discussions.

Method	Data	Accuracy	F1 Rate
Naïve Baes	10 attributes *	0.655	0.611
SVM + GKRBF	10 attributes *	**0.986**	**0.988**
Decision Trees	10 attributes *	0.901	0.922
Neural networks	10 attributes *	0.947	0.956
CNN	*Body* attribute	**0.959**	**0.965**
Lexicon-based Sentiment Analysis	*Body* attribute	0.950	0.811

* *Is_troll*, *Has_verified_email*, *Is_gold*, *Controversiality*, *Comment_karma*, *Link_karma*, *Score*, *Ups*, *Downs*, *Is_mod*.

**Table 14:** Comparison of various approaches to build models for recognition of troll users in online discussions.

Method	Data	Accuracy	Reference
RF	Twitter	0.665	[6]
SMO	Twitter	0.685	[6]
DT	Reddit	0.917	[7]
SVM	Twitter	0.840	[8]
CNN	Twitter	0.740	[8]
BERT	Twitter	0.990	[8]
LSTM	Reddit	0.727	[9]
CNN	Reddit	0.756	[9]
CNN in SA	Hotel reviews	0.823	[11]
LSTM in SA	Hotel reviews	0.850	[11]
RF	Taobao	0.930	[12]
KNN (10)	Kaggle	0.878	[14]
SVM	Reddit	0.986	our
CNN	Reddit	0.959	our
LSA	Reddit	0.950	our

## Data Availability

Machine learning models were trained on dataset “Final_Data_News.csv” available at people.tuke.sk/kristina.machova/useful/. Other type of data—text data (bodies of posts) were used for learning CNN and LSA model. The text data consist of two parts “Is_troll_body.csv” and “Is_not_troll_body.csv” and are available at people.tuke.sk/kristina.machova/useful/.
